# Desmoid tumour: a rare etiology of intestinal obstruction

**DOI:** 10.11604/pamj.2015.22.158.193

**Published:** 2015-10-20

**Authors:** Gaurav Aggarwal, Sumit Shukla, Ankur Maheshwari, Rajkumar Mathur

**Affiliations:** 1 Department of Surgery, MGM Medical College & MY Hospital, Indore, India

**Keywords:** Intestinal obstruction, pandora′s box, desmoid tumour

## Abstract

Intestinal obstruction is a frequently encountered entity in surgical practice. The signs & symptoms, many a times, are suggestive of the level of obstruction, making the diagnosis of obstruction evident. There are various causes of intestinal obstruction which diversify to an enormous extent, stamping on the famous paradigm for the mysterious nature of the abdomen being referred to as the Pandora's Box. In accordance with the above saying, we report a rare case of a desmoid tumour, presenting as intestinal obstruction, which entices us to strongly believe the same.

## Introduction

The abdomen is well known as a “Pandora's Box” and “Intestinal Obstruction” form a major bulk of the disease burden in this “Box”. In accordance with the above saying, we report a rare case of a desmoid tumour, presenting as intestinal obstruction, which entices us to strongly believe the same.

## Patient and observation

**Figure 1:**
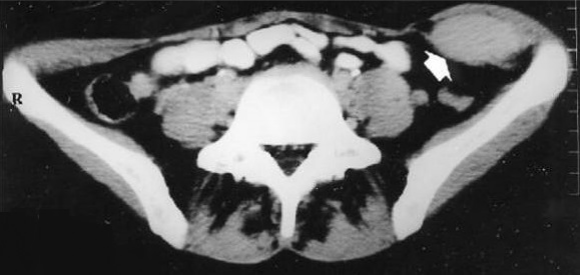
CT Scan of the patient demonstrating the tumour mass

A 28 year old female presented to the emergency department of our hospital, with complaints of early satiety, intermittent episodes of bilious vomiting, a slow growing, painless abdominal mass and an inability to pass motion and flatus since 2 weeks. On examination, a lobulated firm mass extending from the epigastrium to the pubic bones and bilateral lumbar region was found, becoming prominent on straight leg raising test. She also reported a history of appendicectomy and two pregnancies with spontaneous deliveries in 1994 and 1999. Analysed blood parameters were within normal limits. A standing abdominal film showed a soft tissue mass over the whole abdomen. A preoperative CT scan revealed a left lower abdominal wall tumor of unknown dignity (Figure 1).

**Figure 2:**
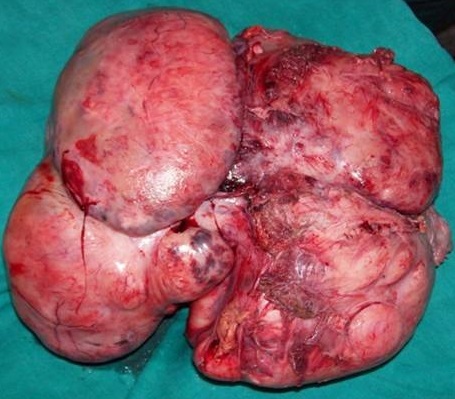
Post operative resected specimen of the tumour mass

The patient was taken up for an emergency laparotomy and intraoperatively, resection of the tumor with excision of the internal oblique abdominal muscle followed, and the defect was subsequently covered with a mesh and strengthened with omentum. Intraoperatively, the anterior rectus sheath was found tightly adherent to the mass. The tumor was also adherent to the parietal peritoneum and bladder on its posterior surface with strong adhesions to the pubic bones inferiorly as well as to the external iliac vein on the right side, which necessitated a vascular repair with prolene 6-0, round bodied, continuous sutures, after excision of the tumor. The tumor was found to cause compression of the intestines & the stomach leading to vomiting & early satiety. The excised tumor weighed 4.6 kg (Figure 2). Subsequent histopathology confirmed the features of a desmoid tumor.

## Discussion

Desmoid tumors are histologically benign fibrous neoplasms originating from the musculoaponeurotic structures throughout the body. The term desmoid, coined by Muller in 1838, is derived from the Greek word desmos, which means “tendon-like”. Although desmoid tumors most commonly arise from the rectus abdominis muscle in postpartum women and in scars due to abdominal surgeries, they may also arise in any skeletal muscle. The tumors tend to infiltrate adjacent muscle bundles, frequently entrapping them and causing their degeneration. Although fixation to musculoaponeurotic structures is apparent, the overlying skin is normal. The myofibroblast is the cell considered to be responsible for the development of desmoid tumors.

Desmoid tumors occur at a rate of 10-15% in patients with Familial Adenomatous Polyposis (FAP), an autosomally inherited disease caused by germline mutations in the APC gene. Sporadic forms have no hereditary background [1]. Desmoid tumors show biallelic APC mutation, with one change usually occurring distal to the second beta-catenin binding/degradation repeat of the gene (3′ to codon 1399) [1, 2]. The relationship between extracolonic manifestations and the site of the APC mutation suggests a specific role of the APC protein in different tissues. However, unknown genetic factors independent of APC may be important in the susceptibility to desmoid tumors in patients with FAP. Catenin and catenin-binding genes have been found to be associated with neoplastic processes in a number of ways. Overall, desmoid tumors are reported to account for 0.03% of all neoplasms. When present in patients with familial polyposis of the colon, the prevalence of desmoid tumors is as high as 13% [3].

CT and MRI are the investigative modalities used to diagnose and for the follow up of desmoid tumors. They can help determine the extent of the tumor and its relationship to nearby structures, especially prior to surgical removal. MRI is superior to CT scan in defining the pattern and the extent of involvement as well as in determining if recurrence has occurred after surgery. The diagnostic test is a biopsy of the tumor. Electron CT scan and MRI are used for the diagnosis and follow-up of desmoid tumors. Microscopy may also be performed. On electron microscopic examination, the spindle cells of desmoid tumors appear to be myofibroblasts. This finding is thought to represent an abnormal proliferation of myofibroblasts, which normally disappear gradually during the later stages of wound healing. Colonoscopy and fundal examination are indicated to investigate for the presence of Gardner's syndrome

Primary surgery with negative surgical margins is the most successful primary treatment modality. Positive margins after surgery reflect a high risk for recurrence [4]. In those patients who refuse surgery or are not surgical candidates, radiation therapy may be used as a treatment of recurrent disease or as primary therapy to avoid mutilating surgical resection; Pharmacologic therapy with anti-estrogens and prostaglandin inhibitors may also be used; In cases of recurrent extra-abdominal desmoid tumors in which surgery is contraindicated or in cases of recurrence, a chemotherapeutic regimen of doxorubicin, dacarbazine, and carboplatin may be effective. Intra-abdominal desmoid tumors as a part of Gardener syndrome may respond to systemic doxorubicin, and ifosfamide can be useful for patients with complications from the tumor [5].

## Conclusion

A high index of suspicion is needed for prompt diagnosis and subsequent timely intervention in cases presenting with intestinal obstruction. Conditions like the one enumerated and discussed above must be borne in mind whilst dealing with unexplained or unremitting abdominal distention, pain or a difficulty in passage of motion-flatus. Thus a high index of suspicion is must for an accurate and timely management even in the rarest of presentation. As the saying goes- *“what the mind knows is what the eyes see”*.
